# Composites of YF_3_: Yb^3+^, Er^3+^, Tm^3+^@C_3_N_4_-Au with near-infrared light-driven ability for photocatalytic wastewater purification†

**DOI:** 10.1039/d4ra07018f

**Published:** 2025-01-09

**Authors:** Zuhuan Long, Yu Gao, Yaojun Zhang, Weili Ma, Jiqi Zheng, Yuxin Liu, Fu Ding, Yaguang Sun, Zhenhe Xu

**Affiliations:** a College of Environment and Chemical Engineering, Dalian University Dalian 116622 Liaoning P. R. China gaoy777@126.com jiqizheng@yeah.net xuzh@syuct.edu.cn; b Key Laboratory of Inorganic Molecule-Based Chemistry of Liaoning Province, Shenyang University of Chemical Technology Shenyang 110142 China dingfu@syuct.edu.cn

## Abstract

Photocatalytic technology for removing organic dye pollutants has gained considerable attention because of its ability to harness abundant solar energy without requiring additional chemical reagents. In this context, YF_3_ spheres doped with Yb^3+^, Er^3+^, Tm^3+^ (YF) are synthesized using a hydrothermal method and are subsequently coated with a layer of graphitic carbon nitride (g-C_3_N_4_) with Au nanoparticles (NPs) adsorbed onto the surface to create a core–shell structure, designated as YF_3_: Yb^3+^, Er^3+^, Tm^3+^@C_3_N_4_-Au (abbreviated as YF@CN-Au). The core–shell composites demonstrate remarkable stability, broadband absorption, and exceptional photocatalytic activity across the ultraviolet (UV) to near-infrared (NIR) spectral range. Notably, by optimizing the amount of Au loaded, excellent methyl orange (MO) degradation rates of 0.068 min^−1^ under UV light and 0.423 h^−1^ under light excitation with *λ* > 420 nm can be achieved. Even under low-energy NIR light (*λ* > 800 nm), a degradation rate of 0.087 h^−1^ was reached, indicating a significantly enhanced degradation effect compared to YF@CN without Au loading. The high performance of the core–shell composite is attributed to its unique structure, which enables efficient transfer of energy and charge carriers, thereby promoting charge separation and suppressing recombination. Furthermore, this article reveals and discusses three distinct photocatalytic mechanisms under UV, visible, and NIR light. This study underscores the considerable promise of core–shell composites in developing efficient g-C_3_N_4_-based broadband photocatalysts, focusing on comprehensive utilization of the solar spectrum through the synergistic effects of plasma and upconversion materials.

## Abstract 2

YF@CN-Au core–shell composites with varying Au NPs amounts were developed to effectively utilize the entire solar spectrum, from UV to NIR, for the photodegradation of MO, thereby significantly enhancing solar energy utilization efficiency.

## Graphical abstract

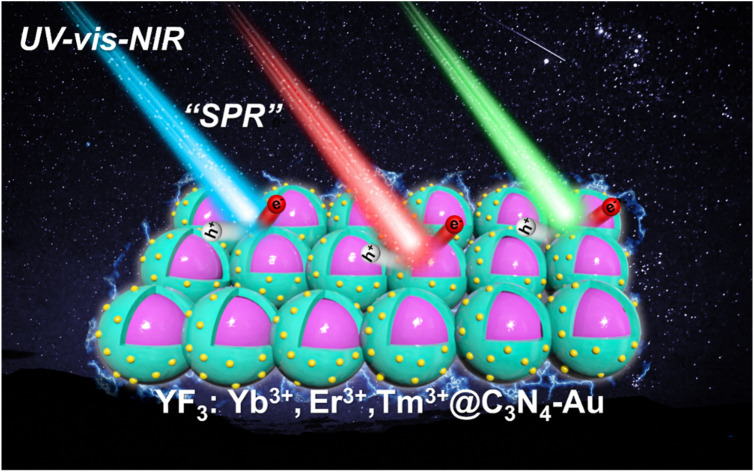

## 1. Introduction

Photocatalysis is recognized as one of the most established strategies for directly harvesting and utilizing solar energy to address industrial wastewater issues, especially those from tannery and textile processing effluents, which are often hazardous to the environment. In the process of wastewater pollutants' photodegradation, semiconductor photocatalysts play a crucial role. An effective photocatalyst should ideally possess not only a wide range of photo-responsiveness but also a minimal recombination rate of photoinduced charge carriers. Currently, a variety of semiconductor photocatalysts, such as TiO_2_, WO_3_, and CdS have been developed. However, certain barriers limit the effectiveness of these semiconductor photocatalysts, including low efficiency, toxicity, high production costs, and poor thermal and chemical stability, all of which significantly hinder their photocatalytic applications. Consequently, developing suitable semiconductor photocatalysts to overcome these challenges has become one of the researchers' most pressing missions.

g-C_3_N_4_ has emerged as a novel semiconductor photocatalyst, owing to its advantages including high chemical inertness, unique layered structure and visible-light responsiveness. Despite these notable properties, several significant shortcomings hinder its photocatalytic efficiency, such as limited surface area and unsatisfactory rate of charge recombination.^1^ Furthermore, the effective excitation wavelength of g-C_3_N_4_ is limited to less than 460 nm, indicating that a considerable portion of solar energy is still not fully utilized. Several approaches have been developed to rectify these shortcomings and broaden the light absorption range, including doping with metal or nonmetal elements, or integrating with other semiconductors to develop heterojunctions.^2^ Amongst them, the surface plasmon resonance (SPR) effect exhibited by coinage metals, including Au and Ag nanoparticles (NPs), has received significant attention.^3–5^ The integration of these materials with semiconductor photocatalysts seeks to address the limitations in the efficiency of photochemical processes through SPR excitation of metals or enhancing charge transfer efficiency.^6,7^ It has been well demonstrated that Au nanoparticles in contact with g-C_3_N_4_ can provide additional active sites for photocatalytic processes and extend the absorption range, and serve as a cocatalyst to suppress charge carrier recombination, thus improving photocatalytic efficiency.^7–10^

The development of photocatalysts with efficient near-infrared (NIR) response spectra to enhance solar energy utilization remains a highly sought-after objective. It is established that upconversion particles doped with rare earth elements (REEs) can effectively convert NIR light into UV and visible light, while also emitting higher-energy photons that can be reabsorbed by adjacent UV and visible light-responsive elements. Moreover, the core–shell structure can not only effectively enhance light absorption but also improve the electron transfer process, thereby increasing the catalytic activity. Studies have demonstrated that core–shell composite materials containing REEs exhibits excellent photocatalytic performance under visible light and NIR irradiation. For instance, the upconversion photocatalyst NaYF_4_@SnO_2_@Ag composite with a core–shell structure exhibited a wide spectral response range from UV to NIR. Benefiting from the unique structure in which the NaYF_4_ core broadens the spectral response range, its RhB photodegradation efficiency can reach 0.56 h^−1^ when *λ* = 980 nm.^11^ The photocatalyst containing a NaGdF_4_:Er^3+^, Yb^3+^ core combined with a bismuth ferrite shell also demonstrated an impressive MO degradation rate of 0.054 h^−1^ when *λ* = 980 nm.^12^ Anwer *et al.* also developed a NaYF_4_: Yb^3+^, Gd^3+^, Tm^3+^@Bi_2_WO_6_ core–shell composite that had high UV-vis-NIR response and showed an improved bisphenol A photodegradation rate of 0.19 min^−1^ under solar illumination.^13^ Such structures were also applied in photocatalytic hydrogen evolution reactions. A CdS@SiO_2_@NaYF_4_:Yb/Tm composite with a dual-core-shell structure exhibited a rate of 74.67 mmol g^−1^ h^−1^ under conditions of *λ* > 420 nm.^14^ These studies confirm that core–shell composite materials containing REEs can effectively broaden the absorption range and improve light utilization efficiency.

Considering the aforementioned points, the systematic integration of lanthanide-doped upconversion particles, g-C_3_N_4_, along with Au NPs results in composite materials, wherein the joint contribution of their individual functions enables comprehensive utilization of solar light for photocatalytic processes. The fabrication of core–shell microspheres by integrating the three aforementioned functional components presents a viable solution to address the existing challenges. Herein, a novel broadband spectrum-responsive core–shell architecture capable of harvesting UV, visible, and NIR light through the integration of these three components was constructed for the first time, and was used for the photodegradation of methyl orange (MO). In this architecture, g-C_3_N_4_ is responsive to UV and part of the visible light spectrum, while Au NPs are capable of absorbing visible light. Additionally, the up-conversion YF_3_ particles doped with Yb^3+^, Er^3+^, Tm^3+^ (YF) can convert NIR to UV and visible light, subsequently utilized by both g-C_3_N_4_ and Au NPs.^15,16^ This process enables effective light harvesting across the UV to NIR regions. Thus the optimized YF@CN-Au composite exhibits remarkable activity across a broad light spectrum, demonstrating not only remarkable performance under UV and visible light but also significant improved effectiveness in the NIR range. Moreover, the photodegradation mechanisms and charge transfer processes that contribute to the elevated photocatalytic performance under UV, visible, and NIR light were well studied and discussed. This study presents a novel approach for developing broadband photocatalysts capable of utilizing full-spectrum solar radiation, and also provide insight into the plasma and upconversion-enhanced photocatalysts.

## 2. Experimental section

### 2.1 Materials

During this experiment, all chemicals used, including chloroauric acid tetrahydrate (AuCl_3_ HCl·4H_2_O), rare-earth nitrate hydrates (Y(NO_3_)_3_·6H_2_O, Yb(NO_3_)_3_·5H_2_O, Er(NO_3_)_3_·5H_2_O, Tm(NO_3_)_3_·5H_2_O), sodium fluoride (NaF), Cyanamide, MO, 1,4-benzoquinone (BQ), disodium ethylenediaminetetraacetate (Na_2_EDTA), and *tert*-butyl alcohol (*t*-BuOH), were purchased from Sigma-Aldrich, were of analytical grade, and were used directly. Throughout the experiment, all water used produced by a Millipore Ultrapure system was absolute pure with a resistivity of 18.2 MΩ cm at room temperature.

### 2.2 Synthesis of spherical YF_3_: Yb^3+^, Er^3+^, Tm^3+^ particles

Typically, add 1.488 mL of 0.50 M Y(NO_3_)_3_ aqueous solution to 15 mL of pure water while stirring magnetically. Then add 0.50 mL of 0.010 M Tm(NO_3_)_3_ solution, 0.10 mL of 0.010 M Er(NO_3_)_3_ solution, and 0.50 mL of 0.50 M Yb(NO_3_)_3_ solution to the mixture. After stirring for 1 h, add 4 mmol of NaF in the solution. Continue stirring for an additional hour. The mixture was placed in a Teflon-lined stainless steel autoclave and heated at 180 °C for 12 h. The obtained product was collected through centrifugation, washed successively with ethanol and water, then air-dried at 80 °C for 12 h. The obtained Yb^3+^, Er^3+^ and Tm^3+^ doped YF_3_ is represented by YF.

### 2.3 Synthesis of core–shell YF_3_: Yb^3+^, Er^3+^, Tm^3+^@C_3_N_4_

For a typical synthesis process, mix 1.0 g of the synthesized YF particles with 5 g of cyanamide, and sonicate at 60 °C for 2.5 h. The obtained mixture was kept stirring for 12 h in a water bath to keep the temperature at 60 °C. The remaining mixture was dried in air after centrifugation to remove the excess cyanamide, and a white solid was obtained. Subsequently, the solid was calcined under a N_2_ atmosphere at 550 °C for 4 h, using a heating rate of 1 °C min^−1^. The obtained yellow powder was washed with water and 0.1 M HNO_3_ for several times, and vacuum-dried at 80 °C overnight after centrifugation. The obtained core–shell YF_3_: Yb^3+^, Er^3+^, Tm^3+^@C_3_N_4_ microspheres is denoted as YF@CN.

### 2.4 Synthesis of core–shell YF_3_: Yb^3+^, Er^3+^, Tm^3+^@C_3_N_4_-Au

A certain amount of AuCl_3_ HCl·4H_2_O along with the produced YF@CN composite were dispersed in 20 mL water, and the mixture was placed into a quartz container. After 2 hours of irradiation with a xenon lamp (300 W), the precipitate was separated through centrifugation, and air-dried at 60 °C for 12 h after washed with ethanol and water to obtain Au NPs modified YF@CN. By adjusting the amount of AuCl_3_ HCl·4H_2_O in the initial raw materials compared to YF@CN, a series of composites can be produced, which are denoted as YF@CN-*x* wt% Au (*x* = 0, 0.5, 1.0, 2.0, and 3.0) based on the mass fraction of Au.

### 2.5 Material characterization

A JEM-2100F JEOL transmission electron microscope (TEM) and high-resolution TEM (HRTEM) under a voltage of 200 kV was used to characterize the microstructures of the products. X-ray diffraction (XRD) patterns were characterized by an X-ray diffractometer (D8 Advance, Bruker) with Cu Kα radiation. The Fourier transform infrared spectra were obtained using the KBr pellet method on a Thermo Scientific Nicolet iS20. UV-vis diffused reflectance spectra were investigated using a UV-vis spectrophotometer (UV2550, Shimadzu) with BaSO_4_ as the standard. X-ray photoelectron spectra (XPS) were characterized by an ESCALAB 220I-XL spectrometer. A CHI 660E workstation was employed to conduct the photoelectrochemical measurements using a three-electrode system. The electrolyte used was a 0.2 M Na_2_SO_4_ aqueous solution that had been purged with N_2_ for 30 min. An Ag/AgCl (3 M KCl) electrode served as the reference electrode, and a Pt wire acted as the counter electrode. To construct the working electrode, 5 mg sample was mixed with 1 mL of dimethylformamide using ultrasound for 30 min. The dispersion was drop-casted on a piece of fluorine-doped tin oxide (FTO) glass, with edges protected by Scotch tape. The uncoated areas were sealed with epoxy resin glue after air drying naturally. The electrochemical impedance spectroscopy (EIS) was performed from 100 kHz to 100 mHz and the transient photocurrent measurements were conducted under 420 nm illumination.

### 2.6 Photocatalytic activity measurements

The photocatalytic performance of YF@CN-Au samples was determined based on the photocatalytic degradation of MO. The reaction took place within a 100 mL quartz reactor that featured circulating cooling. Disperse 10 mg of YF@CN-*x* wt% Au in 20 mL of MO aqueous solution (10 mg L^−1^) and stir in the dark for 1 h to set up an adsorption–desorption balance. The light source was a xenon lamp (300 W, PLS-SXE 300D) with an emission spectrum ranging from 320 to 2500 nm. Appropriate cutoff filters were utilized to allow only the desired wavelength range to pass. A UVREF filter was utilized to capture UV light in the range of 320 nm < *λ* < 400 nm. The UVIRCut420 and UVIRCut475 filters were used to collect light in two visible regions, 420 nm < *λ* < 780 nm and 475 nm < *λ* < 780 nm, respectively. A VisCut800 filter was employed for the NIR region (*λ* > 800 nm). The concentration of MO was determined by extracting 0.5 mL of the solution at various irradiation durations and testing the centrifuged supernatant with a UV-vis spectrophotometer at 462 nm. The photocatalytic stability of the sample was evaluated over 20 MO photodegradation cycles. The sample was extracted *via* centrifugation after each cycle and was washed thoroughly with water several times. The first-order reaction model was adopted to determine the apparent reaction rate constant (*k*) to investigate the kinetics of MO degradation, which can be expressed as:ln(*C*_0_*/C*) = *kt*where *C*_0_ and *C* are the MO concentration before the reaction and at time *t*, respectively. By normalizing *k* based on the mass of the sample, the intrinsic rate constant can be determined. To clarify the active species participated during the photocatalytic reaction, 1 mM BQ, Na_2_EDTA, and *t*-BuOH aqueous solutions were employed as scavengers for superoxide radicals (˙O_2_^−^), holes (h^+^), and hydroxyl radicals (˙OH).

## 3. Results and discussion

**Scheme 1:**
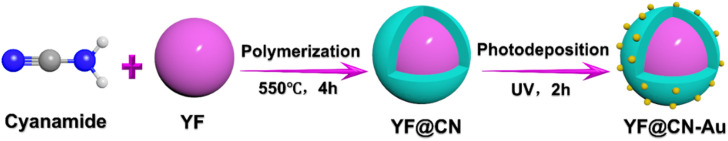
Schematic illustration of the construction of core–shell YF@CN-Au composite.

**Fig. 1:**
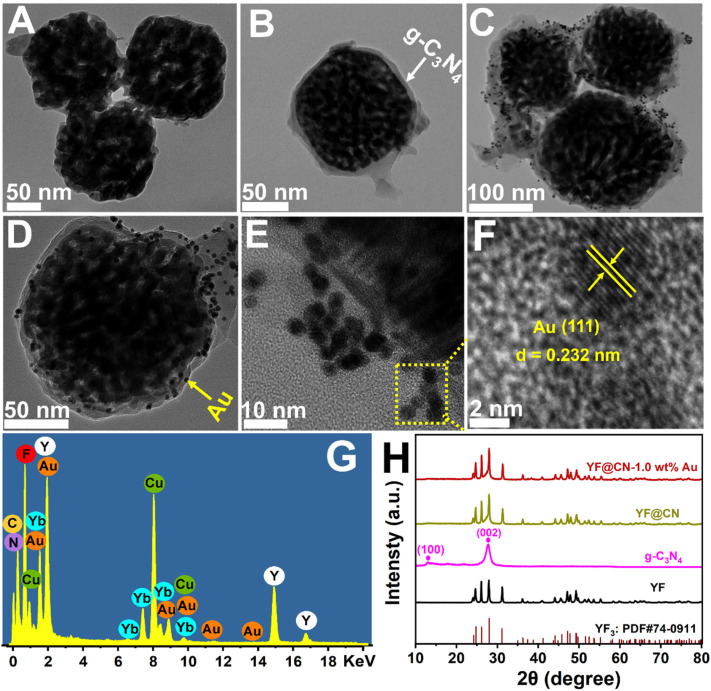
TEM images of (A) YF, (B)YF@CN and (C–E) YF@CN-1.0 wt%Au photocatalysts; (F) the HRTEM image highlighting the lattice fringes of (111) plane of Au; (G) the EDX spectrum of YF@CN-1.0 wt% Au; (H) XRD patterns of YF, g-C_3_N_4_, YF@CN and YF@CN-1.0 wt% Au.

In Scheme 1, the construction process of the core–shell composite YF@CN-Au is graphically demonstrated, which is conducted in three steps: (1) the hydrothermal method is employed to synthesize uniform and well-dispersed YF nanoparticles. The TEM image demonstrates that the diameter of YF nanoparticles is around 180 nm (Fig. 1A). (2) Employing the YF particles as templates, a reaction with cyanamide is performed, followed by calcination to prepare a porous g-C_3_N_4_ shell, leading to the formation of YF@CN with a core–shell microstructure. The TEM image verified that the thickness of the shell is approximately 25 nm, as shown in Fig. 1B. ^17–20^ (3) The core–shell YF@CN microspheres was then mixed with HAuCl_4_ solution, and Ag NPs were deposited onto the surface of YF@CN microspheres through a photo-deposition process. Fig. 1C–E present the TEM images of the YF@CN-1.0 wt% Au sample at varying magnifications, demonstrating the uniform distribution of abundant Au NPs on the surface of YF@CN microspheres, with diameters of around 10–20 nm. The HRTEM image reveals a inter fringe spacing of 0.232 nm, corresponding to the (111) crystal plane of Au (Fig. 1F).^21,22^ The energy-dispersive X-ray spectroscopy (EDX) analysis of a single sphere (Fig. 1G) confirmed the presence of elements C, N, F, Y, Yb, and Au, while Er and Tm were not detected as their concentrations were below the detection limit. The measured Au content in YF@CN-1.0 wt% Au was found to be approximately 0.98%, which is very close to the theoretical value of 1% that was intentionally added during synthesis. Fig. 1H displays the XRD patterns of YF, g-C_3_N_4_, YF@CN, and YF@CN-1.0 wt% Au. The XRD patterns of YF, YF@CN, and YF@CN-1.0 wt% Au reveal that all peaks can be indexed to YF_3_: PDF#74-0911. For g-C_3_N_4_, the peaks observed at 13.1° and 27.8° align with its (100) and (002) planes, respectively. Peaks of g-C_3_N_4_ and YF coexist in XRD pattern of YF@CN composite, confirming that g-C_3_N_4_ successfully encapsulates YF microspheres, and the two compounds were combined successfully. For YF@CN-1.0 wt% Au, as the low content of Au and the overlapping diffraction peaks of Au and YF, the identification of metallic Au through XRD is challenging.^21,23,24^ Due to the low Au content, the peaks of the FTIR spectra (Fig. S1†) of YF@CN and YF@CN-1.0 wt% Au are basically identical, and both are similar to that of g-C_3_N_4_. The sharp peak observed at 808 cm^−1^ is attributed to the stretching vibration of the carbon nitride s-triazine ring, while the significant characteristic peaks in the 1200–1700 cm^−1^ region are associated with the stretching vibrations of the C–N and C=N bonds. The broad bands in the range of 3000 to 3300 cm^−1^ originate from the adsorbed H_2_O and the stretching vibration of N–H bonds.^25,26^ The above characterizations confirm the successful construction of the YF@CN-Au composite with a core–shell structure.

**Fig. 2:**
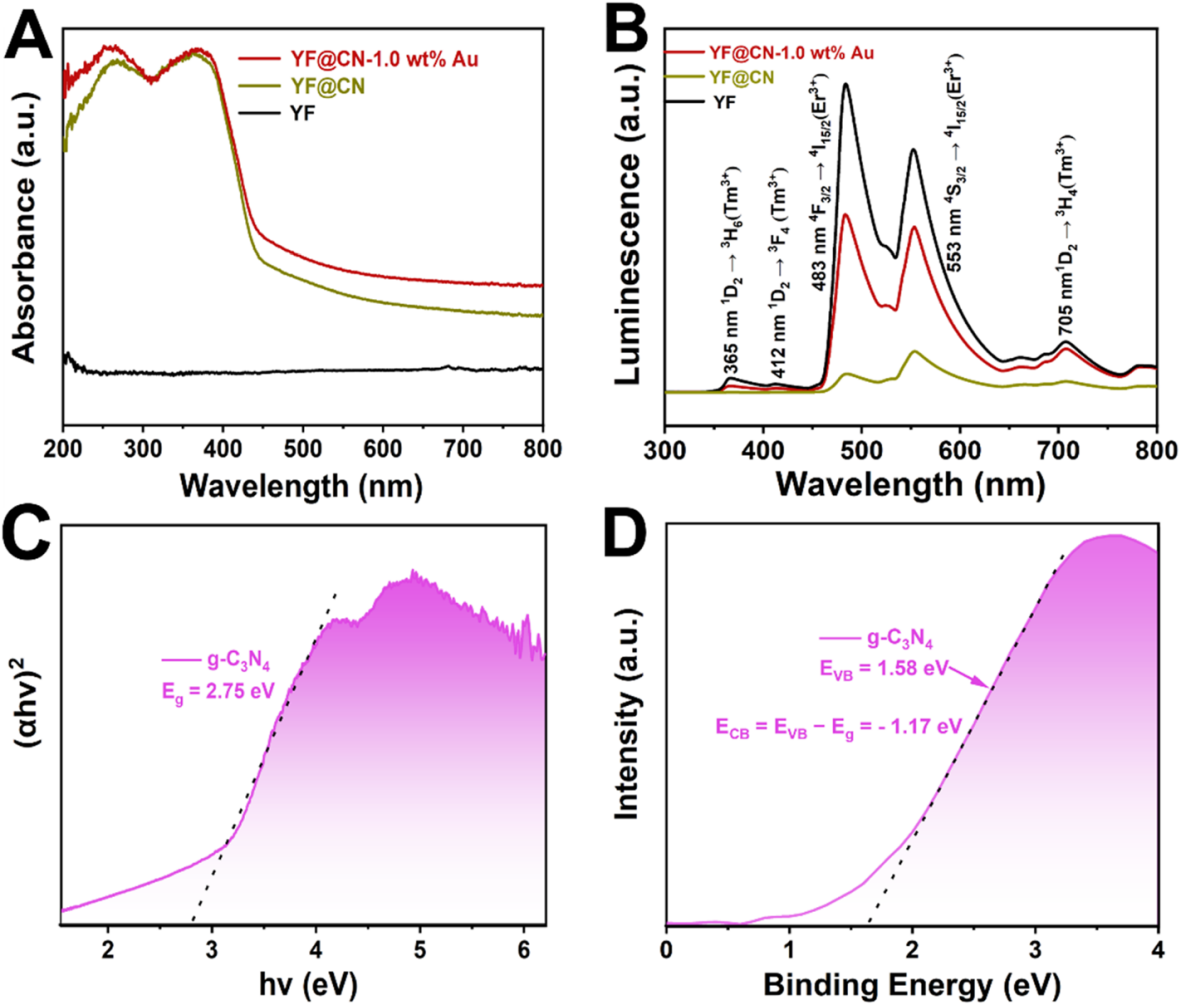
(A) UV-vis spectra of YF, YF@CN and YF@CN-1.0 wt% Au; (B) upconversion photoluminescence spectra of YF, YF@CN and YF@CN-1.0 wt% Au; (C) the Tauc plots and (D) the VB XPS spectrum of g-C_3_N_4._

The UV-vis spectra (Fig. 2A) indicate that pure YF shows no absorption. In the case of YF@CN, with the introduction of the g-C_3_N_4_ shell, a sharp absorption is observed from 460 nm to shorter wavelengths, which corresponds to the characteristic absorption features of g-C_3_N_4_. Upon the deposition of Au NPs, the YF@CN-1.0 wt% Au composite demonstrates an absorption range that extends beyond 460 nm into the visible region, with an increase in absorption intensity towards longer wavelengths. This phenomenon is attributed to the excitation of SPR that occurs when Au NPs are deposited on the YF@CN surface. The up-conversion photoluminescence (PL) spectrum is presented in Fig. 2B. When illuminated by a 980 nm laser, YF microspheres generate emissions with higher energy levels in both UV and visible regions through the conversion of NIR photons. The spectrum indicates that three emission peaks of Tm^3+^ ions are found at 365, 412, and 705 nm, associated with the transition ^1^D_2_ → ^3^H_6_, ^1^D_2_ → ^3^F_4_, ^1^D_2_ → ^3^H_4_. At 483 and 553 nm, the emission peak of Er^3+^ ions correspond to the transition ^4^F_3/2_ → ^4^I_15/2_, ^4^S_3/2_ → ^4^I_15/2_. The UV-vis spectra (Fig. 2A) indicates that the blue UV light emitted by YF will primarily be absorbed by g-C_3_N_4_ because of the *E*_g_ absorption, while the green visible light is expected to be reabsorbed by Au NPs due to the SPR excitation. Once the g-C_3_N_4_ shell has formed, the overall emission is significantly diminished compared to pure YF, indicating that the converted emission is absorbed by g-C_3_N_4_ in YF@CN, which confirms the micro-directional energy transfer from YF to the g-C_3_N_4_ shell. After the introduction of Au NPs, because of the SPR excitation, there is a significant enhancement in the intensity of green visible light centered at 553 nm compared to YF@CN. Thus, the ingeniously designed YF@CN-1.0 wt% Au photocatalyst facilitates the indirect harnessing of NIR light through their conversion to ultraviolet and visible light. The following equation was adopted to calculate the band gaps of g-C_3_N_4_:

1 *αhv* = *A*(*hv*− *E*_g_)^*n*/2^

In this equation, *h* and *ν* denote Planck's constant and the light frequency, respectively; *α* is the absorption coefficient; *A* is a constant; *E*_g_ represents the band gap energy; *n* is a parameter which varies based on specific materials (for g-C_3_N_4_, *n* = 4). Fig. 2C indicates that the deduced *E*_g_ for g-C_3_N_4_ is approximately 2.75 eV, which aligns with previously reported values.^27,28^ In Fig. 2D, the valence band (VB) XPS reveals that the VB edge of g-C_3_N_4_ is 1.58 eV relative to vacuum. Thus for g-C_3_N_4_, the conduction band (CB) energy is calculated by subtracting *E*_g_ from the VB value, resulting in a CB energy of −1.17 eV with respect to vacuum.^29–31^

**Fig. 3:**
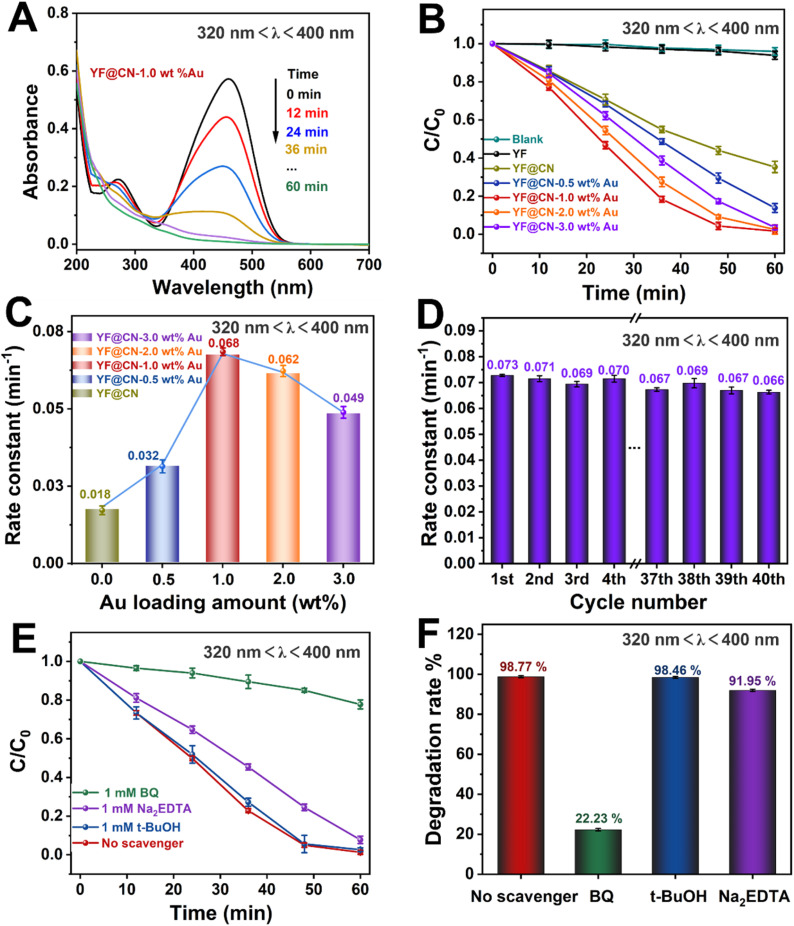
The MO photodegradation activity under UV light (320 nm < *λ* < 400 nm) irradiation: (A) absorption spectra of MO photodegradation for YF@CN-1.0 wt% Au after different irradiation time; (B) the MO photodegradation over YF, YF@CN and YF@CN-*x* wt% Au and (C) the corresponding rate constants; (D) the rate constants of YF@CN-1.0 wt% Au during 40 consecutive cycles, each lasting 1 h; (E) effects of three different scavengers on the MO degradation over YF@CN-1.0 wt% Au and (F) the corresponding MO degradation rates after 60 min.

**Fig. 4:**
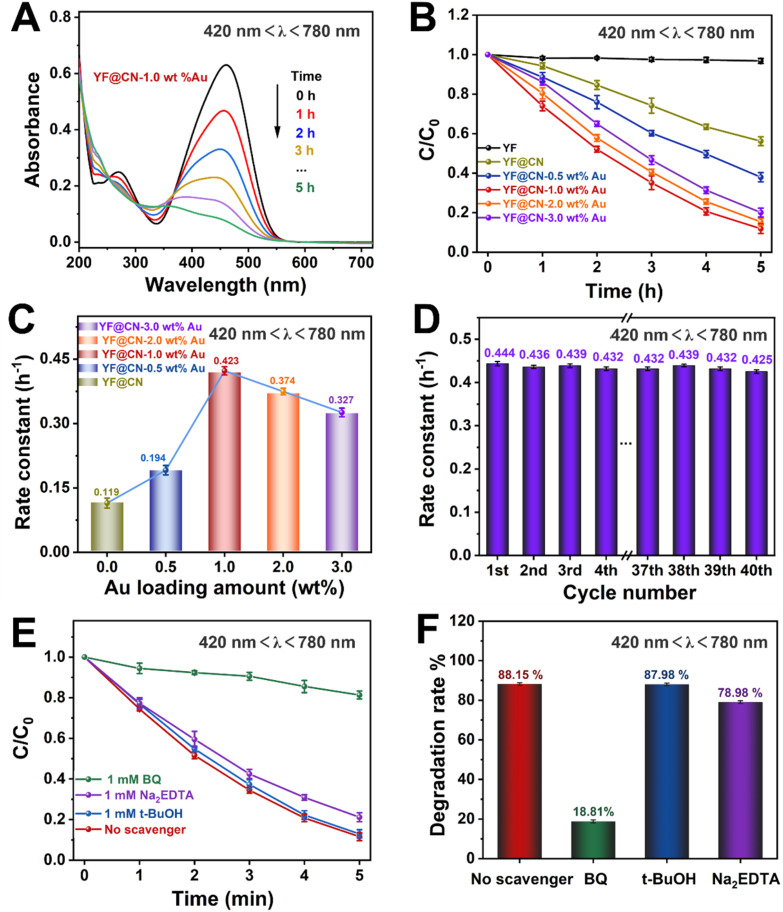
The MO photodegradation activity under visible light (420 nm < *λ* < 780 nm) irradiation: (A) absorption spectra of MO photodegradation for YF@CN-1.0 wt% Au after different irradiation time; (B) the MO photodegradation over YF, YF@CN and YF@CN- *x* wt% Au and (C) the corresponding rate constants; (D) the rate constants of YF@CN-1.0 wt% Au during 40 consecutive cycles, each lasting 5 h; (E) effects of three different scavengers on the MO degradation over YF@CN-1.0 wt% Au and (F) the corresponding MO degradation rates after 5 h.

**Fig. 5:**
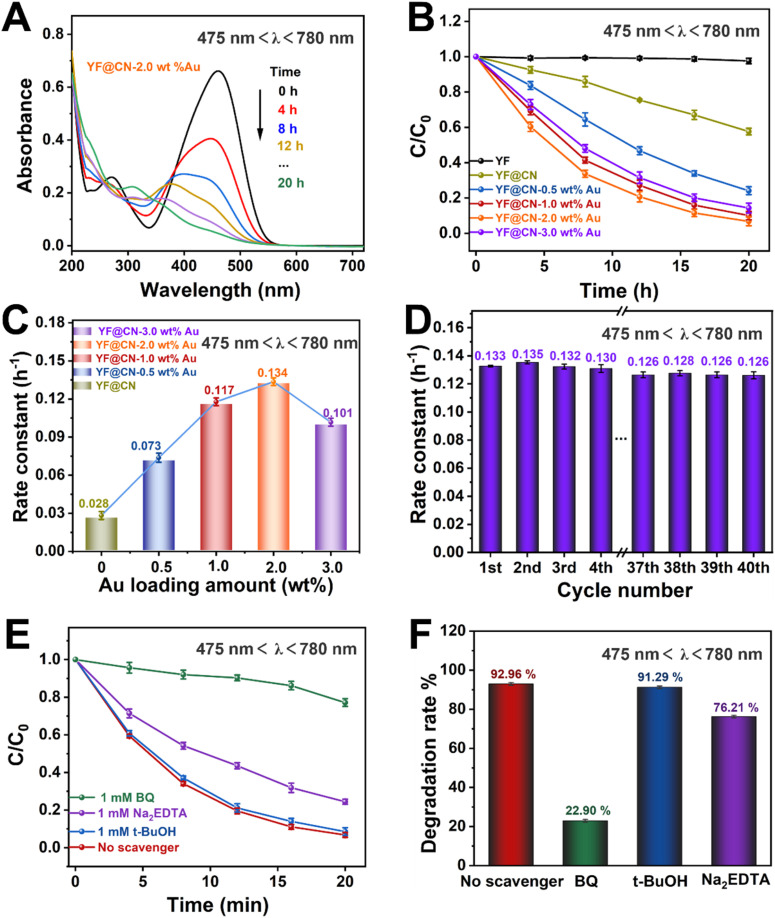
The MO photodegradation activity under visible light (475 nm < *λ* < 780 nm) irradiation: (A) absorption spectra of MO photodegradation for YF@CN-2.0 wt% Au after different irradiation time; (B) the MO photodegradation over YF, YF@CN and YF@CN-*x* wt% Au and (C) the corresponding rate constants; (D) the rate constants of YF@CN-2.0 wt% Au during 40 consecutive cycles, each lasting 20 h; (E) effects of three different scavengers on the MO degradation over YF@CN-2.0 wt% Au and (F) the corresponding MO degradation rates after 20 h.

**Fig. 6:**
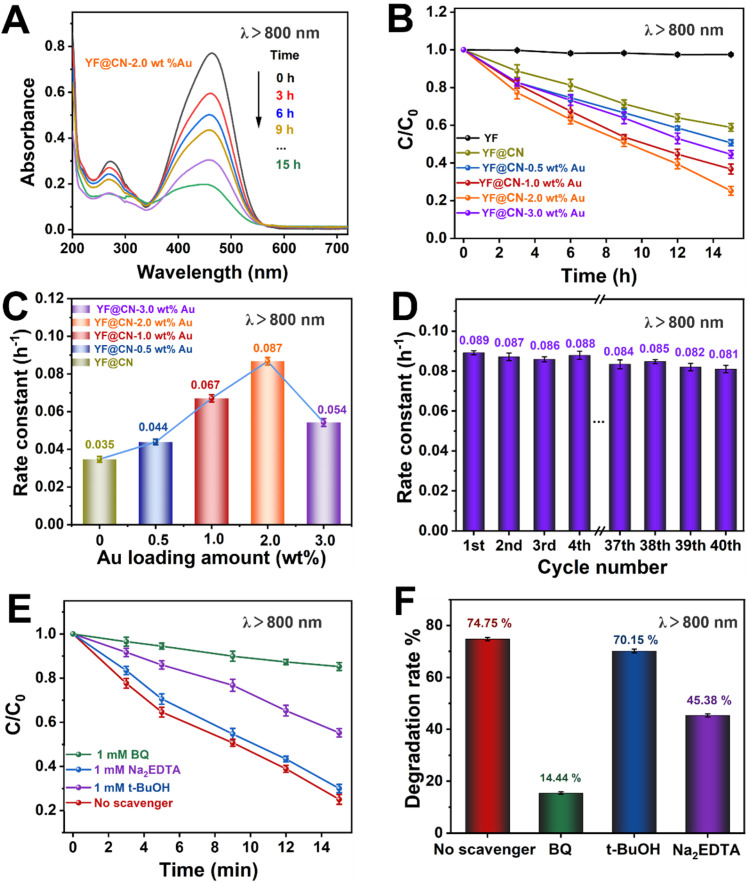
The MO photodegradation activity under NIR light (*λ* > 800 nm) irradiation: (A) absorption spectra of MO photodegradation for YF@CN-2.0 wt% Au after different irradiation time; (B) the MO photodegradation over YF, YF@CN and YF@CN-*x* wt% Au and (C) the corresponding rate constants; (D) the rate constants of YF@CN-2.0 wt% Au during 40 consecutive cycles, each lasting 15 h; (E) effects of three different scavengers on the MO degradation over YF@CN-2.0 wt% Au and (F) the corresponding MO degradation rates after 15 h.

The photocatalytic performance of the obtained photocatalysts was studied under various light conditions using MO as the model pollutant. The performance of the photocatalysts under UV light (320 nm < *λ* < 400 nm), visible light (420 nm < *λ* < 780 nm, 475 nm < *λ* < 780 nm) and NIR (*λ* > 800 nm) is demonstrated in Fig. 3–6, respectively. Given the substantial influence of Au content on the photocatalytic activity, the effect of various Au loadings on photodegradation was systematically examined. As illustrated in Fig. 3A and S2,† the absorption spectrum of MO on YF@CN-1.0 wt% Au displays a characteristic peak at 460 nm, accompanied by a relatively high absorbance. With an increasing duration of light irradiation, the absorbance gradually decreases, indicating the progressive degradation of MO in the solution. After 1 h of UV irradiation, the degradation rates of MO for YF@CN, YF@CN-0.5 wt% Au, YF@CN-1.0 wt% Au, YF@CN-2.0 wt% Au, and YF@CN-3.0 wt% Au are 64.8%, 86.3%, 98.7%, 97.6%, and 95.3%, respectively, as shown in Fig. 3B. Notably, the upconversion material YF shows negligible photocatalytic activity under UV light, as confirmed by the essentially unchanged concentration of MO with pure YF. The loading amount of Au NPs significantly impacts the efficiency of photodegradation. The photocatalytic degradation efficiency reaches its maximum at an optimal loading amount of 1.0 wt% Au NPs. As the Au content is further increased, the photocatalytic activity diminishes. The kinetic study of the MO photodegradation process is illustrated in Fig. 3C. The calculated rate constant follows this order: YF@CN-1.0 wt% Au > YF@CN-2.0 wt% Au > YF@CN-3.0 wt% Au > YF@CN-0.5 wt% Au > YF@CN. The maximum rate constant *k* observed for YF@CN-1.0 wt% Au is 0.068 min^−1^, 3.8 times higher than that of YF@CN. Increasing the loading of Au NPs beyond 1.0 wt% has been shown to reduce photocatalytic activity due to several interrelated factors. Firstly, though low concentrations of Au NPs can enhance photocatalytic activity by improving charge separation efficiency and aiding in visible light harvesting, they may also act as recombination centers, negatively impact the catalytic activity. Thus, excessive amounts of Au NPs may lead to increased exciton recombination rates.^6^ Additionally, the competition for incident light between g-C_3_N_4_ and the supported Au NPs becomes more pronounced. This competition arises from the interband absorption by Au NPs, which limits the light available for the photocatalytic process and ultimately reduces the efficiency.^32,33^ Moreover, the emergence of interface defects due to excessive loading can also lead to a decline in performance.^7^ Consequently, when the loading of Au NPs exceeds 1 wt%, the reaction rate gradually declines, making this the optimal loading level to achieve the highest catalytic performance.

Fig. 3D illustrates the kinetic curve for the photodegradation of MO over 40 cycles in the cyclic test conducted with the YF@CN-1.0 wt% Au. The results indicate that after 40 cycles, the degradation rate of MO exhibited a slight downward trend with minimal fluctuations, demonstrating the excellent stability of the YF@CN-1.0 wt% Au composites. The slight fluctuation may be attributed to the experimental errors, such as fluctuations of the UV-vis spectrophotometer and the inherent variability in the photocatalytic process, which result from changes in light intensity, room temperature, or other environmental conditions during the cycles. EDX (Fig. S3†) analysis after 40 cycles confirmed that the elemental composition remained largely unchanged. The XRD patterns of the sample before and after cycling (Fig. S4†) demonstrate no significant phase changes, as the diffraction peaks remain consistent. Moreover, the TEM image of the recycled catalyst (Fig. S5†) also shows no significant change in microstructure. Collectively, these results indicate that the phase, structure and morphology of YF@CN-1.0 wt% Au remained essentially unaffected after 40 cycles, underscoring its excellent structural stability. To identify the primary active substances involved in the photodegradation of MO, three sacrificial agents (Na_2_EDTA, *t*-BuOH, and BQ) were employed in capture experiments under UV light, utilizing YF@CN-1.0 wt% Au as the photocatalyst. As shown in Fig. 3E and F, the degradation performance of MO shows minimal variation between the blank experiment and the experiments conducted using Na_2_EDTA and *t*-BuOH. However, the presence of BQ significantly reduces the MO degradation efficiency, suggesting that the removal of ˙O_2_^−^ has a considerable impact. Besides, a slight decrease (8.05%) in the degradation rate occurred with the addition of Na_2_EDTA, suggesting the possible participation of h^+^ in this reaction. This result points to the critical importance of ˙O_2_^−^ in MO photodegradation, emphasizing that the superoxide radical serves as a key intermediate, more influential than both holes and ˙OH.

According to UV-vis spectroscopy, the photodegradation process becomes more complex if the reaction is conducted under light with wavelengths exceeding 420 nm, because in this illumination condition, both band gap excitation in g-C_3_N_4_ and SPR excitation in Au NPs occur simultaneously. The absorption spectrum of MO observed under visible light for YF@CN-1.0 wt% Au is comparable to the results obtained under UV light, as illustrated in Fig. 4A and S6.†Fig. 4B indicates that the photocatalytic degradation efficiency of the composite varies with the Au content, and the trend is consistent with that observed under UV irradiation. Under visible light irradiation (420 nm < *λ* < 780 nm), the SPR excitation of Au NPs can generate high-energy electrons, which can be injected into g-C_3_N_4_ to participate in the photocatalytic reaction. Simultaneously, electrons in the CB of g-C_3_N_4_, generated by the band gap excitation, can also potentially transfer to the Au NPs, which is the same as what occurs under UV light exposure.^34^ Thus, the incorporation of Au NPs into g-C_3_N_4_ results in a notable enhancement of the rate constant, increasing from 0.119 h^−1^ for YF@CN to 0.423 h^−1^ for YF@CN-1.0 wt% Au, as shown in Fig. 4C. The excellent stability of the YF@CN-1.0 wt% Au under this irradiation condition was demonstrated through its kinetic curves over 40 cycles of MO photodegradation (Fig. 4D), where only a slight decrease was observed. In addition, active species trapping experiments were carried out using YF@CN-1.0 wt% Au as the photocatalyst to investigate the active species participating in MO photodegradation when 420 nm < *λ* < 780 nm (Fig. 4E and F). In comparison to the scavenger-free system, adding BQ reduces the photodegradation activity of MO by 70.06%, and the presence of Na_2_EDTA decreases the activity by 9.17%. Adding *t*-BuOH exerts minimal influence on photodegradation rate. These findings suggest that h^+^ is involved in the process, while ˙O_2_^−^ has a more significant role.

To investigate the underlying mechanisms for each light source and evaluate how the SPR effect of Au NPs contributes to the photodegradation of MO under visible light, comparative experiments were conducted under visible light irradiation (475 nm < *λ* < 780 nm). Under this condition, the band gap excitation of g-C_3_N_4_ is effectively suppressed. As illustrated in Fig. 5A and S7,† the absorption spectra of MO on YF@CN-2.0 wt% Au at 475 nm demonstrate that the absorbance of MO gradually decreases upon irradiation, indicating a reduction in its concentration. Fig. 5B shows that YF@CN-2.0 wt% Au exhibits the highest degradation efficiency, followed by YF@CN-1.0 wt% Au, which significantly outperforms YF@CN without Au, clarifying that the SPR effect of Au NPs is essential for enhancing visible light-driven photocatalytic performance. While it is theoretically anticipated that the reaction rate will increase with higher Au content, the experimental results reveal that *t* YF@CN-2.0 wt% Au exhibits the highest *k* value, approximately 4.8 times greater than that of YF@CN. It is noteworthy that under visible light (475 nm < *λ* < 780 nm), the light absorption capability of Au particles becomes more critical, as they effectively absorb visible light and promote the separation of photogenerated charge carriers, while the role of g-C_3_N_4_ diminishes due to its weaker light absorption capacity in the visible spectrum. As a result, the optimal Au loading ratio increases from 1% under UV light to 2% under visible light to maximize catalytic activity. Further increasing the Au content lead to a decrease in degradation efficiency (Fig. 5C), because excess Au NPs may introduce defects at g-C_3_N_4_/Au NPs interface between, which can serve as centers for carrier recombination.^7,35,36^Fig. 5D presents stability of the YF@CN-2.0 wt% Au photocatalyst under visible light irradiation (475 nm < *λ* < 780 nm). The degradation rate for MO removal by the catalyst over 40 photoreaction cycles, conducted under identical conditions, remains approximately 0.125 h^−1^, indicating the excellent durability and structural stability. Furthermore, we elucidated the degradation reaction mechanism of YF@CN-2.0 wt% Au through free radical trapping experiments. In the presence of *t*-BuOH, the photocatalytic performance remained largely unchanged in comparison to the condition when no scavenger was added, as demonstrated in Fig. 5E and F, confirming that ˙OH do not participate in the reactions. In contrast, the degradation rate decreased significantly by 70.04% with the addition of BQ, and by 16.75% after the addition of Na_2_EDTA. These results highlight the important roles of ˙O_2_^−^ and h^+^ during the photocatalytic degradation process.

Even under low-energy NIR light irradiation (*λ* > 800 nm), YF@CN-Au composites still demonstrate photocatalytic ability, as indicated by the decreased absorbance of MO over time shown in Fig. 6A and S8.†Fig. 6B further demonstrates a significant reduction in the MO concentration over time. Since pure g-C_3_N_4_ or Au NPs cannot be excited by NIR light, they are unable to photodegrade MO under such irradiation.^37^ Therefore, the observed photodegradation activity originates from the presence of upconversion materials. It is well established that upconversion materials, in this case YF, can emit high-energy light including UV and visible light when irradiated by light with lower energy (NIR). These emitted lights are anticipated to be reabsorbed by both Au NPs and g-C_3_N_4_ for the further catalytic reaction.^38,39^ Thus YF plays a vital role in the photodegradation process when illuminated by NIR light. The photocatalytic activity of the samples in this condition is ranked as follows YF@CN-2.0 wt% Au (0.087 h^−1^) > YF@CN-1.0 wt% Au (0.067 h^−1^) > YF@CN-3.0 wt% Au (0.054 h^−1^) > YF@CN-0.5 wt% Au (0.044 h^−1^) > YF@CN (0.036 h^−1^), which makes 2.0 wt% the optimal loading amount of Au NPs (Fig. 6C). Notably, the degradation rate of YF@CN-2.0 wt% is 2.4 times greater than that of YF@CN. Moreover, only slight attenuation (from 0.089 to 0.081 h^−1^) in degradation efficiency was observed during 40 cycles for YF@CN-2.0 wt% Au (Fig. 6D), confirming its high structural stability. The control experiments using three scavengers were also conducted to assess the role of free radical species and to elucidate the photocatalytic reaction mechanism (Fig. 6E and F). Under NIR light (*λ* > 800 nm), the degradation rate decreases from 74.75% to 14.44% with the addition of BQ and from 74.75% to 45.38% with Na_2_EDTA, while the introduction of *t*-BuOH had a negligible effect. These findings point to the crucial role of ˙O_2_^−^ and h^+^ free radicals in the degradation pathway.

**Fig. 7:**
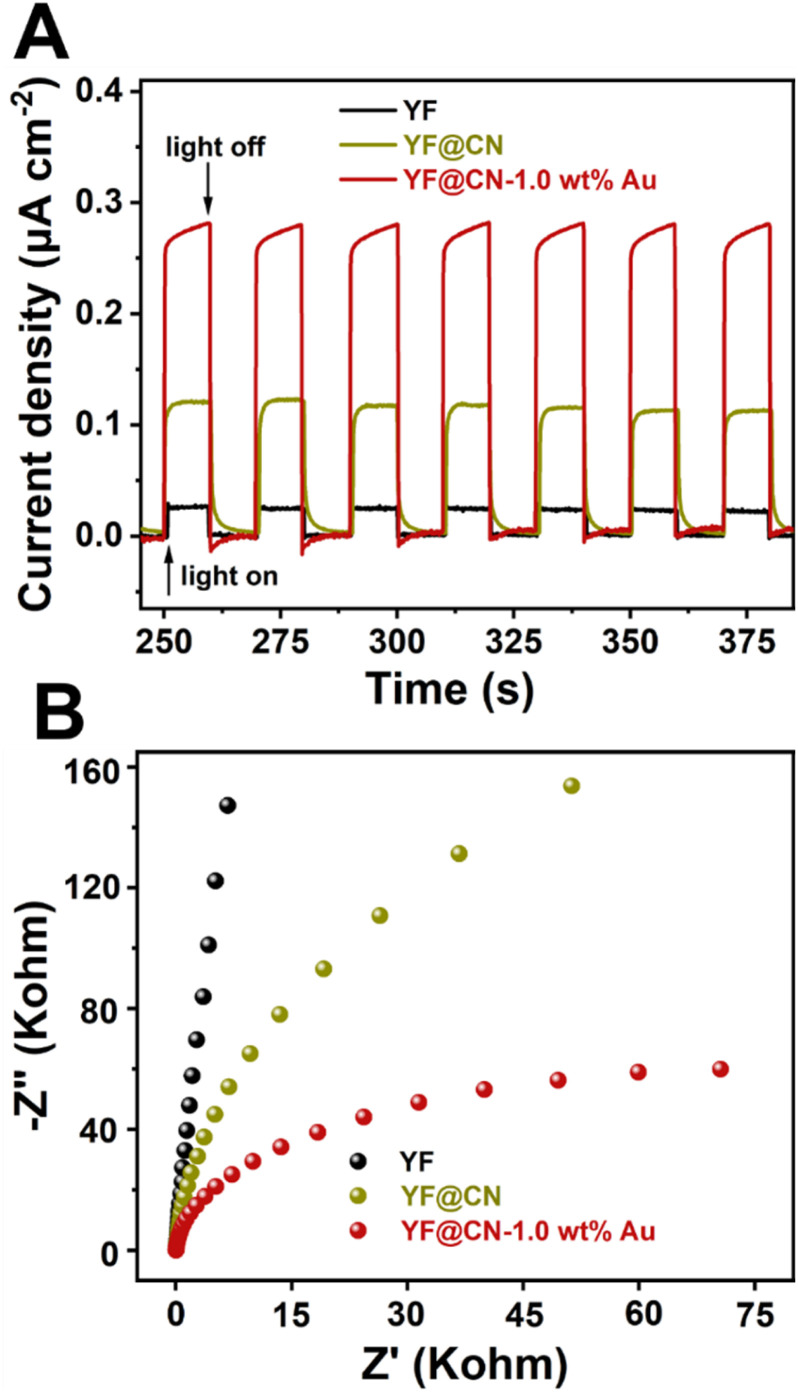
(A) The transient photocurrent density response and (B) the Nyquist plots of YF, YF@CN and YF@CN-1.0 wt% Au.

Photoelectrochemical measurements were conducted to assess the transient photocurrent response of YF, YF@CN, and YF@CN-1.0 wt% Au. As illustrated in Fig. 7A, transient photocurrent measurements were performed during photoswitching cycles to evaluate the carrier generation and transfer efficiency of the catalyst. The results indicate that the saturated photocurrent density remains stable when the light is illuminated. When the light is turned off, the current drops rapidly to nearly zero. The photocurrent density of YF@CN-1.0 wt%Au (approximately 0.28 μA cm^−2^) is about 2.3 times higher than that of YF@CN (approximately 0.12 μA cm^−2^), while YF shows the lowest photocurrent response. This observation suggests that YF itself lacks photocatalytic activity, while the introduction of Au NPs enhances the separation of photogenerated charges, resulting in enhanced photocatalytic activity.^40–42^ Notably, the EIS testing further corroborates these findings. As shown in Fig. 7B, the arc radius for YF@CN-1.0 wt% Au is smaller than that of both YF@CN and YF, confirming reduced charge transfer resistance attributed to the presence of Au NPs, thereby facilitating interfacial charge transfer and suppressing the recombination between photogenerated holes and electrons. This result reveals that the YF@CN-1.0 wt% Au composite accelerates the separation and transfer of photogenerated carriers, enhancing the degradation efficiency of MO.

**Fig. 8:**
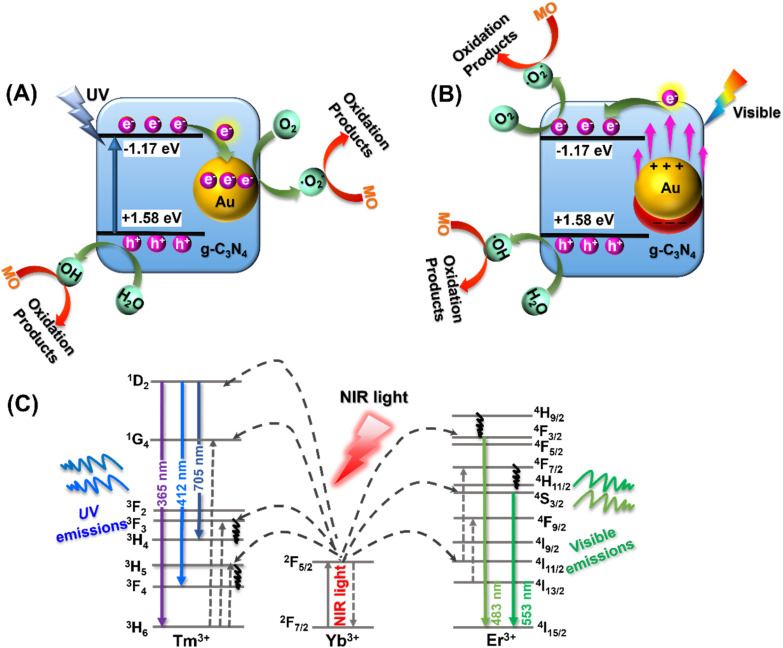
The proposed MO photodegradation mechanisms catalyzed by YF@CN-Au nanocomposite under (A) UV light (320 nm < *λ* < 400 nm), (B) visible light (475 nm < *λ* < 780 nm), and (C) NIR region (*λ* > 800 nm).

Based on the above optical characterization and analysis, it appears that the MO photocatalytic degradation using the YF@CN-Au photocatalyst under different light irradiation may follow distinct pathways. To elucidate the underlying mechanisms associated with different illumination conditions, Fig. 8 presents three potential schematics of the charge transfer process. The degradation of MO by YF@CN-*x* wt% Au under UV light irradiation is illustrated in Fig. 8A. When g-C_3_N_4_ is irradiated by UV light, electrons in its VB are excited and promoted to its CB, resulting in the formation of holes in the VB. The excited electrons in g-C_3_N_4_ CB then migrate to the deposited Au NPs that are in close proximity to g-C_3_N_4_, thereby enhancing the interfacial charge migration and minimizing the recombination of photogenerated carriers.^43^ Electrons transferred to Au NPs react with O_2_ to form ˙O_2_^−^, while the h^+^ that remains in the g-C_3_N_4_ VB can react with H_2_O or OH^−^ to produce ˙OH. Based on the aforementioned scavenging experiments, ˙O_2_^−^ and h^+^ are identified as the active species in the oxidative decomposition of MO, converting it into oxidation products in aqueous solution.

In the process of photodegradation under visible light, when the wavelength surpasses 475 nm (Fig. 8B), the excitation of the *E*_g_ in g-C_3_N_4_ diminishes. Therefore, the photodegradation process relies predominantly on the photogenerated charge carriers generated by the SPR of Au.^44^ High-energy electrons induced by SPR can penetrate the energy barrier and enter the CB of g-C_3_N_4_, where these injected hot electrons further interact with nearby O_2_, generating ˙O_2_^−^ radicals. Meanwhile, h^+^ present in Au NPs can also contribute to the decomposition process of MO.^44,45^ When the wavelength of the irradiation is above 420 nm, both of the aforementioned electron transfer pathways coexist, resulting in a complex mixed mechanism. In this case, ˙O_2_^−^ radicals are simultaneously generated on Au NPs and in the CB of g-C_3_N_4_, leading to an enhanced degradation efficiency of MO. Under NIR (*λ* > 800 nm) irradiation, the upconversion and energy transfer processes are illustrated by a schematic diagram shown in Fig. 8C. Yb^3+^ ions typically act as sensitizers, undergoing energy level transitions from ^2^F_7/2_ to ^2^F_5/2_ when absorbing external energy, results in the formation of an absorption band. Then the energy transfer from Yb^3+^ to the activators Tm^3+^ and Er^3+^ ions leads to their excitation, which subsequently leads to the emission of UV and visible light, as confirmed by the PL spectrum shown in Fig. 2B. The upconverted emissions generated by Tm^3+^ have shorter wavelengths, thus they can be reabsorbed by the g-C_3_N_4_ shell. This reabsorption allows electrons to be excited and promoted to the g-C_3_N_4_ CB, which then generate the degradation-active substance ˙O_2_^−^ radicals for the oxidation of MO.^40,44^ Similarly, the visible light emitted from Er^3+^ ions with a longer wavelength induces SPR in Au NPs, which generates hot electrons and injects them into g-C_3_N_4_, further reducing O_2_ and produce ˙O_2_^−^. The remaining holes in g-C_3_N_4_ VB and Au NPs also contribute to photocatalysis. Thus, through the energy upconversion process from Yb^3+^ to g-C_3_N_4_ and Au NPs, The utilization efficiency of NIR light has significantly increased, and the photocatalytic activity of the YF@CN-Au composite under simulated sunlight can be greatly enhanced.^46^

## 4. Conclusion

In summary, a straightforward method was employed to design and synthesize YF@CN-Au core–shell composites with varying amounts of Au NPs. These photocatalysts demonstrate enhanced photocatalytic activity over a broad spectral range, including UV, visible, and NIR light. Notably, the degradation efficiency of MO is significantly improved, and the catalysts exhibit stable durability. Furthermore, we investigated the catalytic mechanism under three types of illumination: under UV light irradiation, the catalytic effect is attributed to the excitation of the g-C_3_N_4_ band gap; under visible light irradiation, it arises from the SPR excitation of Au NPs; in the NIR region, the upconversion material YF emits UV and visible light, which is subsequently reabsorbed by Au NPs and g-C_3_N_4_ for further excitation, thus enhancing the degradation effect. The synthesized photocatalyst exhibits a high solar energy utilization rate, low Au loading, and remarkable stability. Together, these factors contribute to a favorable economic and environmental profile for industrial applications. This research offers new strategies for optimizing sunlight utilization and effectively degrading organic pollutants, providing a promising approach for addressing environmental contamination.

## Data availability

The data supporting this article have been included as part of the ESI.†

## Conflicts of interest

The authors declare no competing interest.

## Supplementary Material

RA-015-D4RA07018F-s001
