# Supplementary material for: Composites of YF3: Yb3+, Er3+, Tm3+@C3N4-Au with near-infrared light-driven ability for photocatalytic wastewater purification
Source: RSC Adv. 2025 Jan 9;15(2):764–76. doi: 10.1039/d4ra07018f (PMC11715219; doi:10.1039/d4ra07018f)
Supplement: RA-015-D4RA07018F-s001 [file RA-015-D4RA07018F-s001.pdf]

Supplementary Information (SI) for RSC Advances.
This journal is © The Royal Society of Chemistry 2025

Supplementary Information

Composites of YF_3: Yb^{3+}, Er^{3+}, Tm^{3+}@C_3N_4-Au with near-infrared light-driven ability for photocatalytic wastewater purification

Zuhuan Long[a], Yu Gao[a,*], Yaojun Zhang[a], Weili Ma[a], Jiqi Zheng[a,*], Yuxin Liu[a], Fu Ding,[b,*], Yaguang Sun[b], Zhenhe Xu[*,a]

[a]College of Environment and Chemical Engineering, Dalian University, Dalian 116622, Liaoning, P. R. China;

[b]Key Laboratory of Inorganic Molecule-Based Chemistry of Liaoning Province, Shenyang University of Chemical Technology, Shenyang, 110142, China.

*Correspondence: gaoy777@126.com (Y. Gao); jiqizheng@yeah.net (J. Zheng); dingfu@syuct.edu.cn (F. Ding); xuzh@syuct.edu.cn (Z. Xu);

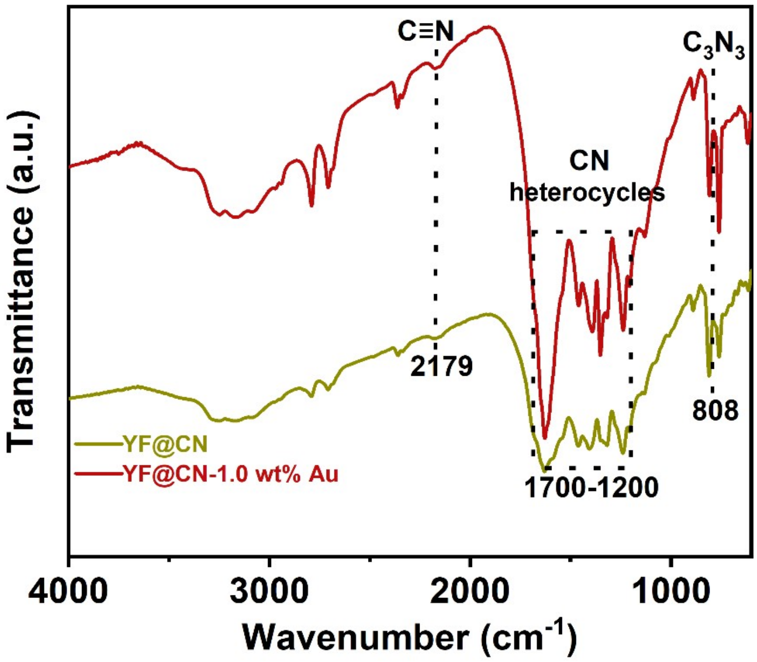

Figure S1. FT-IR spectrum of YF@CN and YF@CN-1.0 wt% Au.

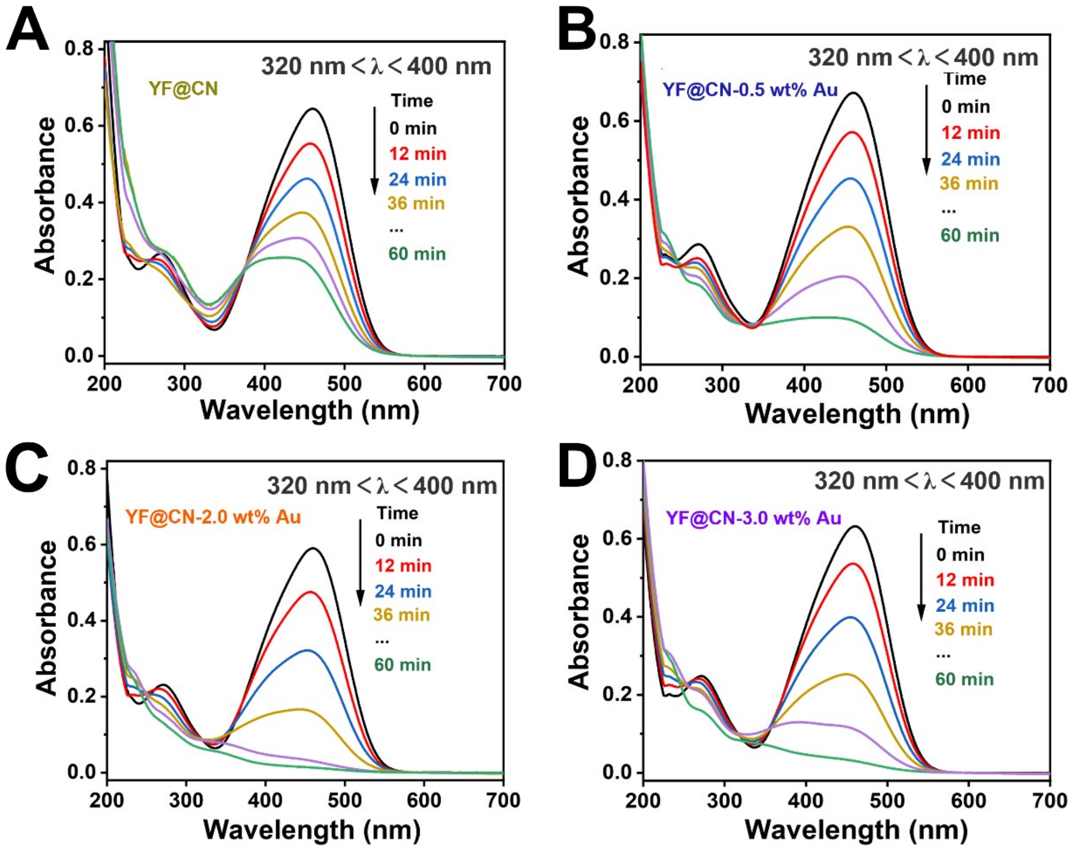

Figure S2. The absorption spectra of MO photodegradation for various mass fractions of Au after different irradiation times under UV light (320 nm< **λ** < 400 nm) irradiation are presented as follows: (A) YF@CN, (B) YF@CN-0.5 wt% Au, (C) YF@CN-2.0 wt% Au and (D) YF@CN-3.0 wt% Au.

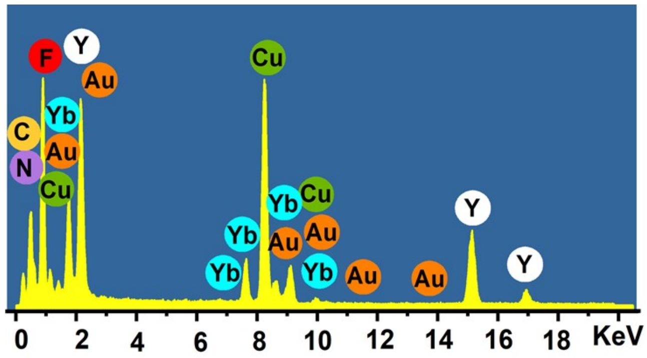

Figure S3. EDX spectrum of YF@CN-1.0 wt%Au after 40 consecutive cycles under UV light (320 nm< λ < 400 nm) irradiation.

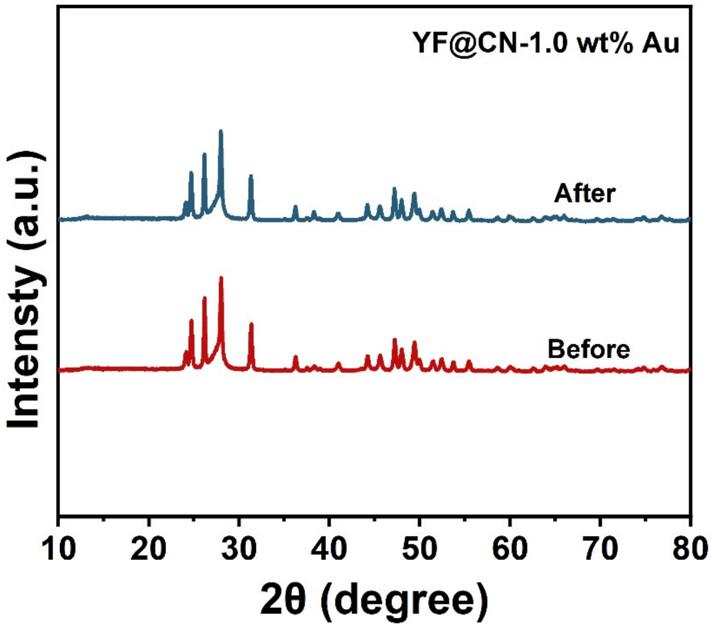

Figure S4. XRD patterns of YF@CN-1.0 wt%Au before and after 40 consecutive cycles under UV light (320 nm< λ < 400 nm) irradiation.

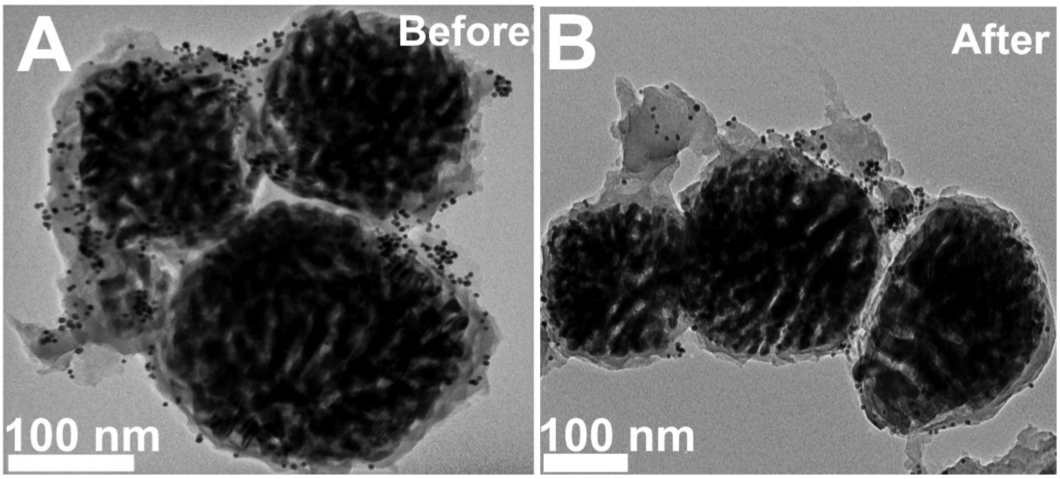

Figure S5. TEM images of YF@CN-1.0 wt%Au before (A) and after (B) 40 consecutive cycles under UV light (320 nm< λ < 400 nm) irradiation.

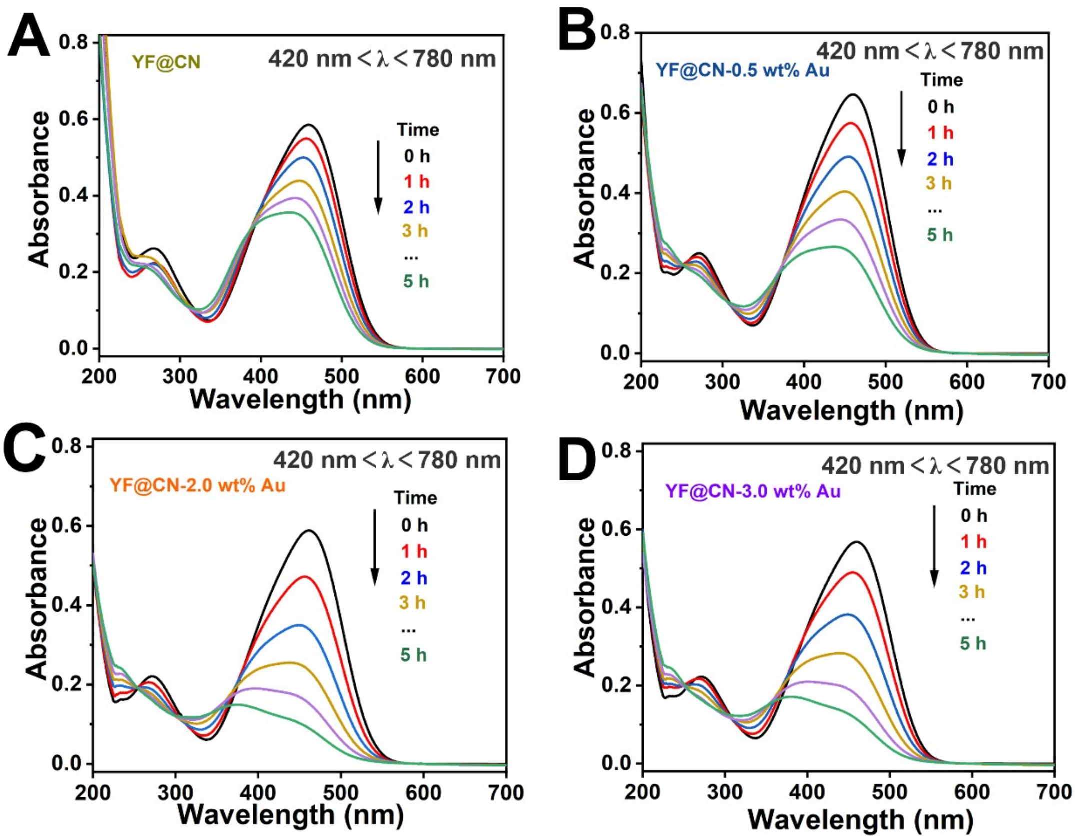

Figure S6. The absorption spectra of MO photodegradation for various mass fractions of Au after different irradiation times under visible light (420 nm< **λ** < 780 nm) irradiation are presented as follows: (A) YF@CN, (B) YF@CN-0.5 wt% Au, (C) YF@CN-2.0 wt% Au and (D) YF@CN-3.0 wt% Au.

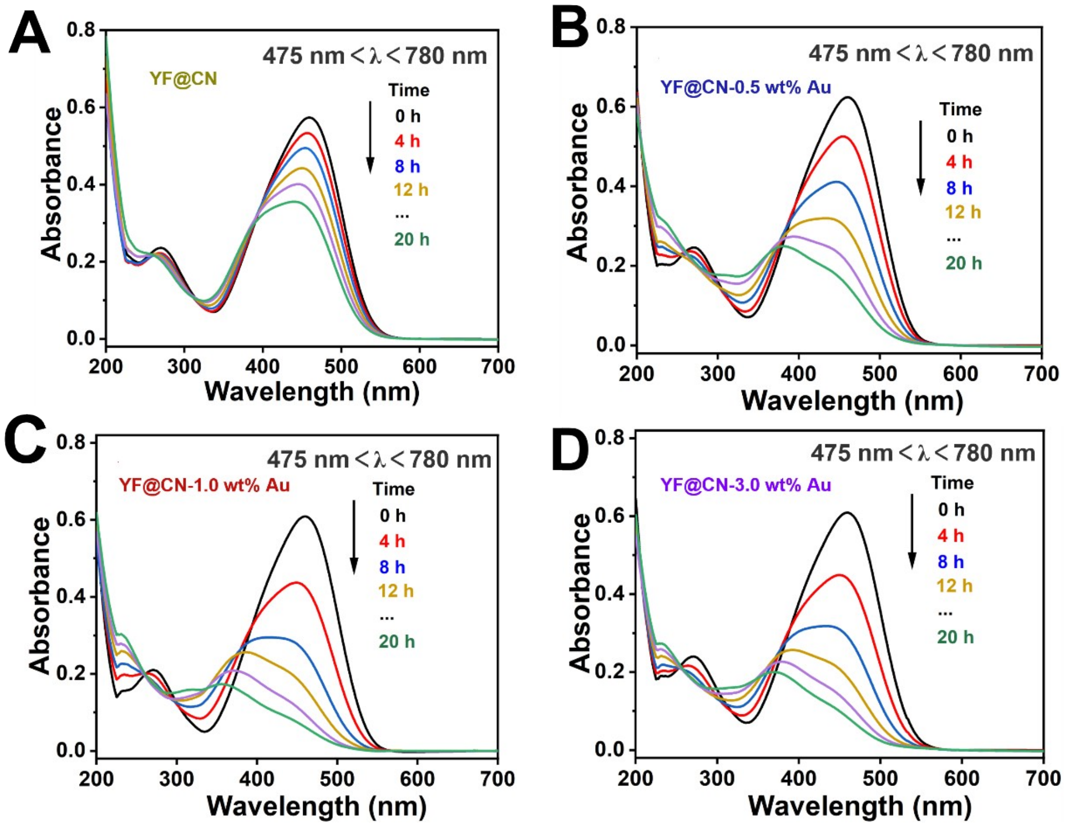

Figure S7. The absorption spectra of MO photodegradation for various mass fractions of Au after different irradiation times under visible light (475 nm< **λ** < 780 nm) irradiation are presented as follows: (A) YF@CN, (B) YF@CN-0.5 wt% Au, (C) YF@CN-1.0 wt% Au and (D) YF@CN-3.0 wt% Au.

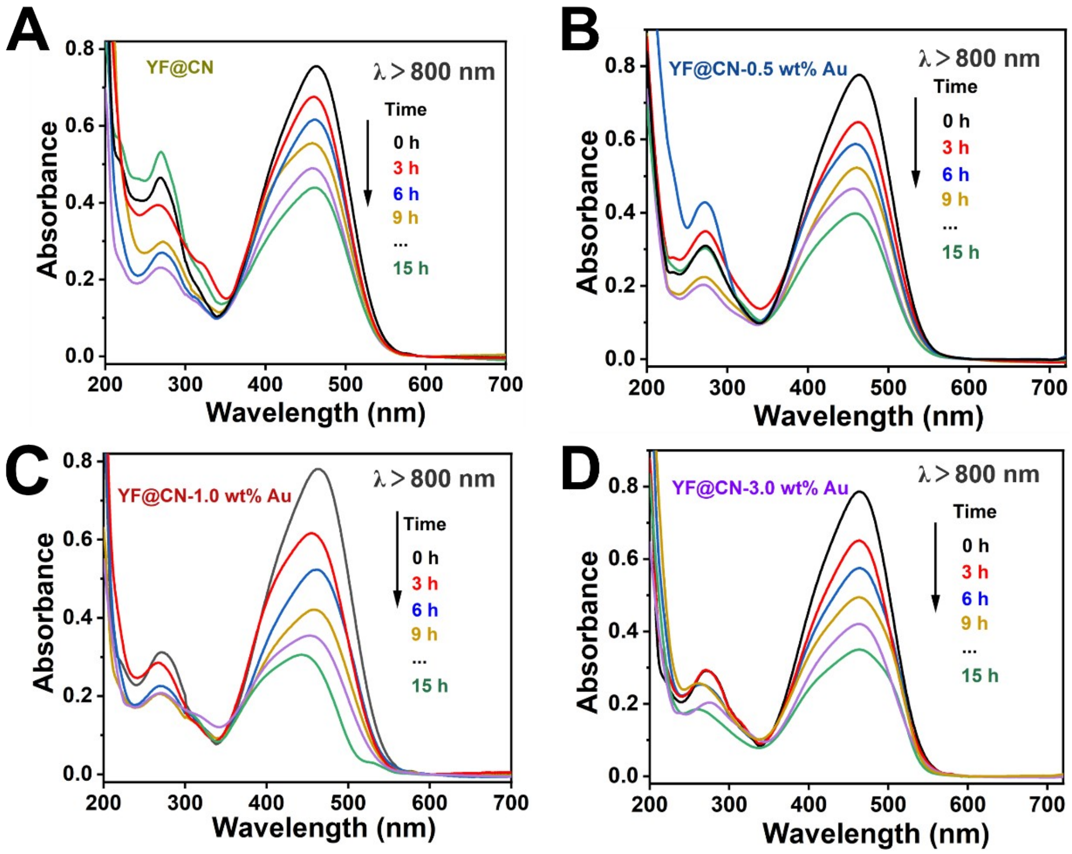

Figure S8. The absorption spectra of MO photodegradation for various mass fractions of Au after different irradiation times under NIR light (**λ** > 800 nm) irradiation are presented as follows: (A) YF@CN, (B) YF@CN-0.5 wt% Au, (C) YF@CN-1.0 wt% Au and (D) YF@CN-3.0 wt% Au.
